# Misaponin B Induces G2/M Arrest, Cytokinesis Failure and Impairs Autophagy

**DOI:** 10.1155/2020/5925094

**Published:** 2020-02-07

**Authors:** Gunho Won, Ji Hoon Jung, Eun Jung Sohn, Ji Eon Park, Hyungjin Kim, Hyo-Jung Lee, Bum Sang Shim, Sung-Hoon Kim

**Affiliations:** College of Korean Medicine, Kyung Hee University, Seoul 02447, Republic of Korea

## Abstract

Saponins are a group of naturally occurring plant glycosides with the features of their strong foam-forming properties and multibiological effects such as antitumor activity. Though Misaponin B, one of the triterpenoid saponins from *Madhuca longifolia*, is known to have spermicidal and antioxidant activity, the other biological activities have been never reported so far. Thus, in the present study, the antitumor mechanism of Misaponin B was investigated in A549 and AsPC-1 cancer cells. Misaponin B exerted significant cytotoxicity in A549, H460, SKOV3, and AsPC-1 cancer cells. Among them, A549 and AsPC-1 cells were more susceptible to Misaponin B. Misaponin B induced G2/M arrest and cytokinesis failure and increased the expression of LC3B and p62 with autophagic vacuoles and GFP-LC3 punctae in A549 and AsPC-1 cells. Furthermore, Misaponin B suppressed autophagy flux in A549 cells transfected by GFP-mRFP-LC3 constructs by showing merged yellow color by autophagy flux assay. Overall, our findings provide evidences that Misaponin B induces G2M arrest and impairs autophagy in A549 and AsPC-1 cells.

## 1. Introduction

Though the war on cancer actively began with the National Cancer Act of 1971, cancer death rate still remains high so far [1]. Among many cancer therapies, the efficacy of chemotherapy in neoplastic patients is extremely limited, due to drug resistance and toxic side effects of drugs [2–4]. Thus, natural antitumor agents such as curcumin [5], decursin [6, 7], and gallotannin are on the spotlight because of their weak toxicity and synergistic efficacy with classical anticancer drugs.

Autophagy is known as a self-degradative process in removing misfolded or aggregated proteins, clearing damaged organelles, such as mitochondria, endoplasmic reticulum and peroxisomes, and intracellular pathogens [8, 9]. When autophagy, an intracellular degradation system, is activated in the cells, double-membrane autophagic vesicles (autophagosomes) formed to surround cytoplasm and/or cytoplasmic constituents and proteins are then fused with lysosomal vesicles to degrade the contents of the autophagic vesicle for complete autophagy [10–13]. In contrast, impaired autophagy, not leading to autophagolysosome, usually contributes the type 2 cell death known as autophagic cell death in all eukaryotic cells [14–17]. It is well documented that autophagic cell death plays an important role in the suppression of malignant tumors [17–19].

Emerging evidences reveal that some saponins including dioscins, saikosaponins, julibrosides, soy saponins, ginseng saponins, and avicins [20, 21] have antitumor effects in several cancers via regulation of apoptosis, autophagy, tumor microenvironment, and cell cycle arrest [22].

Misaponin B (MSB) is a novel triterpenoid saponin isolated from *Bassia longifolia* L. or *Madhuca longifolia* L. [23]. However, its biological effects were never reported except its spermicidal and antioxidant activity and our poster presentation [24]. Thus, in the present study, we report the underlying antitumor mechanism of Misaponin in A549 and ASPC1 cells in association with autophagic cell death.

## 2. Materials and Methods

### 2.1. Isolation of Misaponin B from *Madhuca longifolia* Seeds

The dried seed kernels (100 g) of *M. longifolia* were extracted with 80% aqueous methanol (MeOH, 500 mL × 3), and the extracted solution was filtered and evaporated at 40°C. The concentrate was transferred in water (200 mL) and consecutively extracted with ethyl acetate (EtOAc, 200 mL × 3) and *n*-butanol (*n*-BuOH, 170 mL × 3). The *n*-BuOH layer was concentrated to acquire residues of the *n*-BuOH fraction (MLB, 11.4 g). The MLB fraction was poured to a Diaion HP column (5 cm × 20 cm), eluting water (500 mL) and MeOH (800 mL). The MeOH solution was concentrated at 40°C to give a crude saponin fraction (MLCS, 8.2 g). A part of MLCS fraction (2 g) was subjected to silica gel column chromatography (c.c., 5 cm × 10 cm) and eluted with CHCl_3_–MeOH–H_2_O (6 : 4:1, 1250 mL) to provide seven fractions (MLCS-1 to MLCS-7). Fraction MLCS-4 (375 mg) was applied to the silica gel c.c. (3 cm × 10 cm) and eluted with CHCl_3_–MeOH–H_2_O (8 : 5:1, 450 mL) to provide five fractions (MLCS-4-1 to MLCS-4-5) along with Misaponin B (MLCS-4-3, 187 mg). Misaponin B was identified on the basis of spectroscopic analyses such as NMR, IR, and MS and stored at −20°C. The purity was >90%, as assessed by HPLC.

### 2.2. Cell Culture

Human A549, H460, SKOV3, and AsPC-1 cancer cells were purchased from American Type Culture Collection (ATCC, USA). The cells were cultured in RPMI1620 (WELGENE, Cat. LM 011-01, Korea) medium supplemented with 10% FBS and 1% antibiotic-antimytotic solution (WELGENE. Cat. LS203-01, Korea) at 37°C and CO_2_.

### 2.3. Cell Viability Assay

The cytotoxic effects of Misaponin B in A549, H460, SKOV3, and AsPC-1 cancer cells were evaluated by 3-(4, 5-dimethylthiazol-2-yl)-2,5-diphenyltetrazolium bromide (MTT) assay. Cells were seeded onto 96-well microplates at a density of 1 × 10^4^ cell/well and treated with various concentrations of Misaponin B (0, 7.5, 15, 30, 45, or 60 *μ*M) for 24 h. The cells were incubated with 3-(4,5-dimethylthiazol-2-yl)-2,5-diphenyl tetrazoliumbromide (1 mg/mL) (Sigma Chemical Co., USA) for 1 h, and then optical density (OD) was measured using a microplate reader (Molecular Devices Co., USA) at 570 nm. Cell viability was calculated as a percentage of viable cells in Misaponin B-treated group versus untreated control.

### 2.4. Cell Cycle Analysis

To perform cell cycle analysis, A549 and ASPC1 cells were treated with Misaponin B for 24 h, collected, and fixed in 75% ethanol. The fixed cells were then incubated at 37°C with 0.1% RNase A in PBS for 2 h and suspended in PBS containing 50 *μ*g/mL PI for 30 min at room temperature. The stained cells were analyzed for DNA content in the FACSCalibur (Becton Dickinson, Franklin Lakes, NJ, USA) using the Cell Quest program (Becton Dickinson, USA).

### 2.5. Quantitative Real-Time PCR (qRT-PCR) and Reverse Transcription PCR (RT-PCR)

Total RNA was isolated from A549 and AsPC-1 cells with QIAzol (Invitrogen, USA). A reverse transcription kit (Promega, USA) was used to construct the template cDNA. qRT-PCR was performed with the LightCycler480 instrument (Roche Applied sciences, USA). GAPDH served as normalization control. Primer sequences used for PCR are as follows: LC3B forward 5′-AGACCTTCAAGCAGCGCCG-3′, LC3B reverse 5′-ACACTGACAATTTCATCCCG-3′; miR1290 forward 5′-GAGCGTCACGTTGATCACGTTGACACT-3′, reverse 5′-TTGAGCATCCCTGATCCA-3′; glyceraldehyde-3-phosphate dehydrogenase (GAPDH) forward 5′-TATAAATTGAGCCCGCAGCC-3′, GAPDH reverse 5′-TTCCCGTTCTCAGCCTTGAC-3′. The PCR was performed as follows: 5 min at 95°C, 30 cycles of 95°C for 20 sec, 55°C for 20 sec, 72°C for 40 sec, and 5 min incubation at 72°C. The PCR products were loaded onto 1% agarose gel electrophoresis and visualized by loading STAR solution (Dyne-bio, Korea) and UV illumination.

### 2.6. Western Blot Analysis

A549 and AsPC-1 cells were lysed in RIPA buffer (3 M, Lot#R1140305) and were incubated on ice for 1 h. After centrifugation, the protein contents were measured by using a Bio-Rad DC protein assay kit II. Proteins were separated by electrophoresis on 8% or 12% SDS-PAGE and electrotransferred onto a Hybond ECL transfer membrane. The membrane was blocked with 5% nonfat skim milk and probed with primary antibodies for LC3B, p62 (Cell Signaling Tech., USA), and *β*-actin (Santa Cruz Biotechnologies, USA), followed by incubating with horseradish peroxidase- (HRP-) conjugated secondary antibodies. Protein expression was detected by using an enhanced chemiluminescence (ECL) system (Amersham Pharmacia, USA).

### 2.7. Immunofluorescence

A549 and AsPC-1 cells were seeded on a chamber slide (Nalge Nunc. LAB-TEK. 177399, USA) and were then treated with 15 *μ*M or 30 *μ*M of Misaponin B for 24 h. After washing, the cells were fixed with 4% paraformaldehyde in PBS for 20 min, washed twice with PBS for 10 min, and permeabilized by using 0.5% of Triton X-100 for 5 min at room temperature, followed by PBS with 2% bovine serum albumin (BSA) (AMRESCO. Albumin, Bovine. CAS# 9048-46-8, USA) for 30 min. The anti-α-tubulin antibody was incubated. After washing twice with DPBS, A549 and AsPC-1 cells were incubated with Alexa 546 conjugated goat anti-rabbit IgG antibody for 1 h. The chamber slide was mounted with a VECTASHIELD mounting medium with DAPI. Samples were imaged under a confocal fluorescence microscope (FV1000, Olympus, Japan).

### 2.8. Autophagy Flux Assay Using RFP-GFP-LC3 Construct

A549 cells incubated on a cell culture slide (SPL. Lot No. BA5A09A, Korea) were transfected with green fluorescent protein-labeled LC3 (GFP-LC3) or red-green fluorescent protein-labeled LC3 (RFP-GFP LC3) using X-treme GENE HP DNA Transfection Reagent (Roche, Lot. 11062400, Switzerland). The fluorescent color was analyzed in Misaponin B-treated A549 cells for 24 h by fluorescence microscopy.

### 2.9. Transmission Electron Micrograph (TEM)

A549 cells were treated with Misaponin B for 24 h and fixed for 12 h in 2% glutaraldehyde - paraformaldehyde in 0.1 M phosphate buffer (pH 7.4) and washed in 0.1 M phosphate buffer and were postfixed with 1% OsO_4_ in 0.1 M phosphate buffer for 2 h and dehydrated in an ascending gradual series (50∼100%) of ethanol and infiltrated with propylene oxide. After pure, fresh resin embedding and polymerization at 65°C on a electron microscope oven (TD-700, DOSAKA, Japan) for 24 h, thick sections were cut and stained with toluidine blue (Sigma, T3260, USA) and were double marked with 6% uranyl acetate (EMS, 22400 for 20 min) and lead citrate (for 10 min) for contrast staining. All the pieces were observed by transmission electron microscopy (JEM-1011, Japan) at the voltage of 80 kV. The grid is removed, blotted with a filter paper, and positioned onto a drop of 2% uranyl acetate for 15 sec. After removing the excess uranyl acetate, the EM grid was photographed for transmission electron microscopy (JEM-1011, Japan). A549 cells were randomly selected and imaged at 4,000x or 10,000x magnification by transmission electron microscopy.

### 2.10. Statistical Analysis

GraphPad Prism 5.0 software was used for statistical analyses. Data were analyzed using one-way ANOVA or two-way ANOVA. *p* < 0.05 was considered statistically significant.

## 3. Results

### 3.1. Misaponin B Induced Cytotoxicity and G2/Arrest in A549 Cells

**Figure 1:**
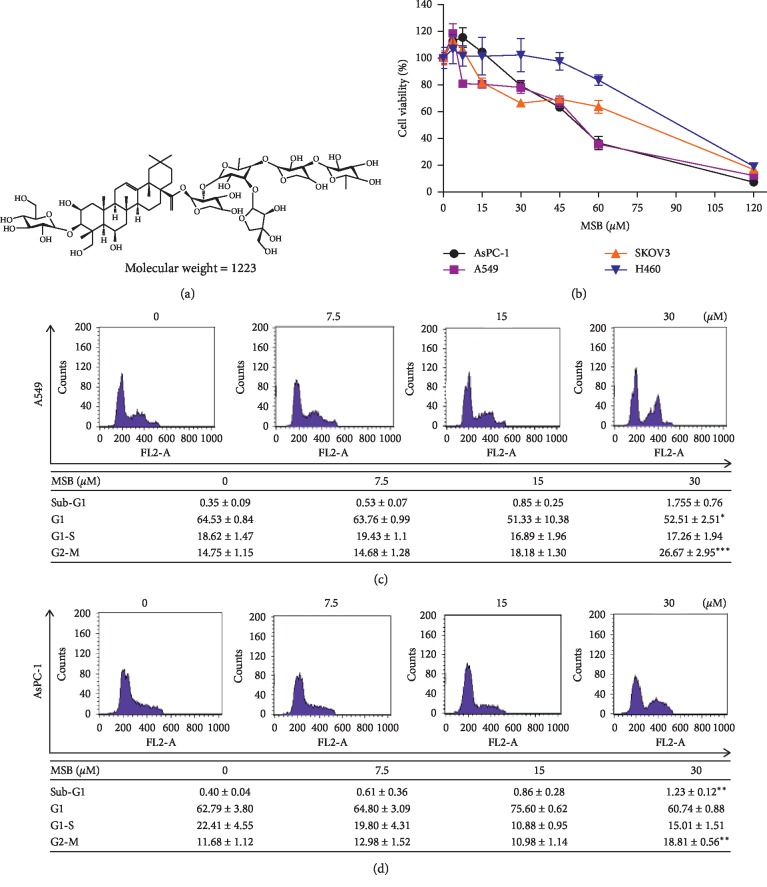
Misaponin B exerts cytotoxicity in several cancer cells, such as A549, H460, AsPC-1, and SKOV3 cancer cells and induces G2/M arrest in A549 cells and AsPC-1 cells. (a) Chemical structure of Misaponin B (MSB). (b) Cytotoxicity of Misaponin B in several cancer cells. MTT assay was used to evaluate the cytotoxicity of Misaponin B in A549, H460, AsPC-1, and SKOV3 cells. (c, d) Effect of Misaponin B on G2/M arrest in A549 and AsPC-1 cells by FACS analysis. Data are represented as means ± SD of two independent experiments. ^*∗*^*p* < 0.05; ^*∗∗*^*p* < 0.01; ^*∗∗∗*^*p* < 0.001 vs untreated control.

To investigate the cytotoxic effect of Misaponin B (Figure 1(a)), different concentrations of Misaponin B (7.5, 15, 30, 45, 60, 120 *μ*M) were added for 24 h in NSCLC (A549 and H460), pancreatic cancer AsPC-1, and ovarian cancer SKOV3 cells. Misaponin B reduced viability in a concentration-dependent manner in A549, H460, AsPC-1, and SKOV3 (Figure 1(b)). A549 and AsPC-1 cells were most susceptible to Misaponin B among the treated cancer cells. Next, cell cycle analysis was performed following PI staining by FACS. Here, Misaponin B induced G2/M arrest in A549 and AsPC-1 cells (Figures 1(c) and 1(d)).

### 3.2. Misaponin B Induced Cytokinesis Failure in A549 Cells

**Figure 2:**
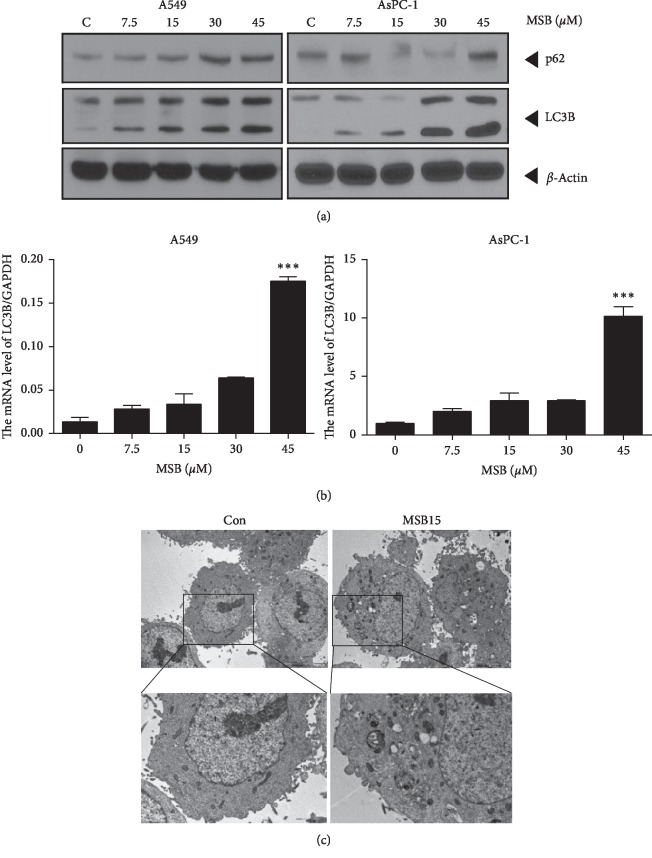
(a) Effect of Misaponin B on LC3B conversion and p62 at the protein level in A549 and AsPC-1 cells. Western blot analysis of A549 and AsPC-1 cells were treated with Misaponin B (7.5 to 45 *μ*M) for 24 h and subjected to western blotting (*n* = 2). (b, c) Effect of Misaponin B on LC3B at mRNA level. A549 and AsPC-1 cells were treated with Misaponin B for 24 h and subjected to RT-PCR analysis. (d) Transmission electron microscopy (TEM) images showing autophagic vacuoles in Misaponin B-treated A549 cells. Misaponin B-treated A549 cells were photographed by TEM (*n* = 3).

Cytokinesis was evaluated in Misaponin B-treated A549 cells. Briefly, A549 cells were treated with different concentrations (15, 30 *μ*M) of Misaponin and stained with DAPI for immunofluorescence. As shown in Figures 2(a) and 2(b), a fraction of *α*-tubulin was localized to the centrosomes, but not moved to the cytokinesis for mitotic cell division.

### 3.3. Misaponin B Increased Accumulation of Autophagic Marker LC3B in a Concentration-Dependent Manner in A549 and AsPC-1 Cells

To evaluate the effect of Misaponin B on autophagy, western blotting was conducted in A549 and AsPC-1 cells. Misaponin B increased LC3B conversion and p62/SQSTM1 accumulation at the protein level in a concentration-dependent fashion in the A549 or AsPC-1 cells (Figure 2(a)). Consistently, qRT-PCR analysis revealed that the mRNA expression level of LC3B was increased in Misaponin B-treated A549 or AspC-1 cells (Figures 2(b) and 2(c)). TEM is one of the standard methods to detect autophagy [25]. TEM observation revealed that the number of autophagosomes was observed in the cytoplasm of A549 cells treated by Misaponin B (Figure 2(d)).

### 3.4. Misaponin B Increased the Number of LC3-Positive Fluorescent Punctae and the Formation of Autophagosomes in Misaponin B-Treated A549 Cells

**Figure 3:**
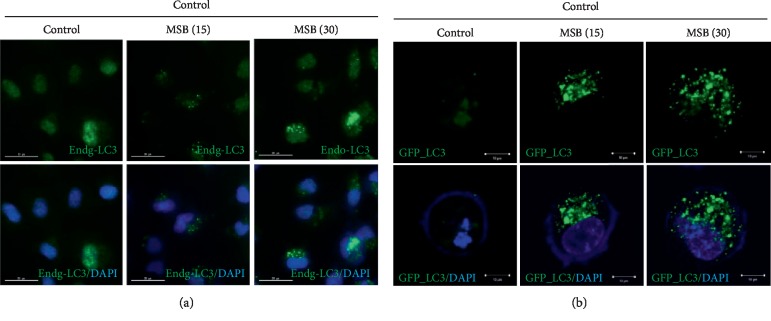
Misaponin B increased the number of LC3-positive fluorescent punctae and autophagosomes in A549 cells. (a) Misaponin B increased the punctae of endogenous LC3B in A549 cells. Immunostaining was performed with LC3 antibody in Misaponin B-treated A549 cells. (b) Misaponin B increased the autophagosome formation in GFP-LC3 expressing A549 cells. After transfection with GFP-LC3 construct, A549 cells were exposed to Misaponin B for 24 h and then visualized by confocal microscopy (*n* = 2).

Misaponin B increased the punctae stained with endogenous LC3 (endog LC3) and DAPI in A549 cells compared to untreated controls (Figure 3(a)). Consistently, the number of green GFP-LC3 autophagosomes was significantly increased in Misaponin B-treated A549 cells compared to untreated control (Figure 3(b)).

### 3.5. Mi-saponin B Inhibited Autophagy Flux in A549 Cells

**Figure 4:**
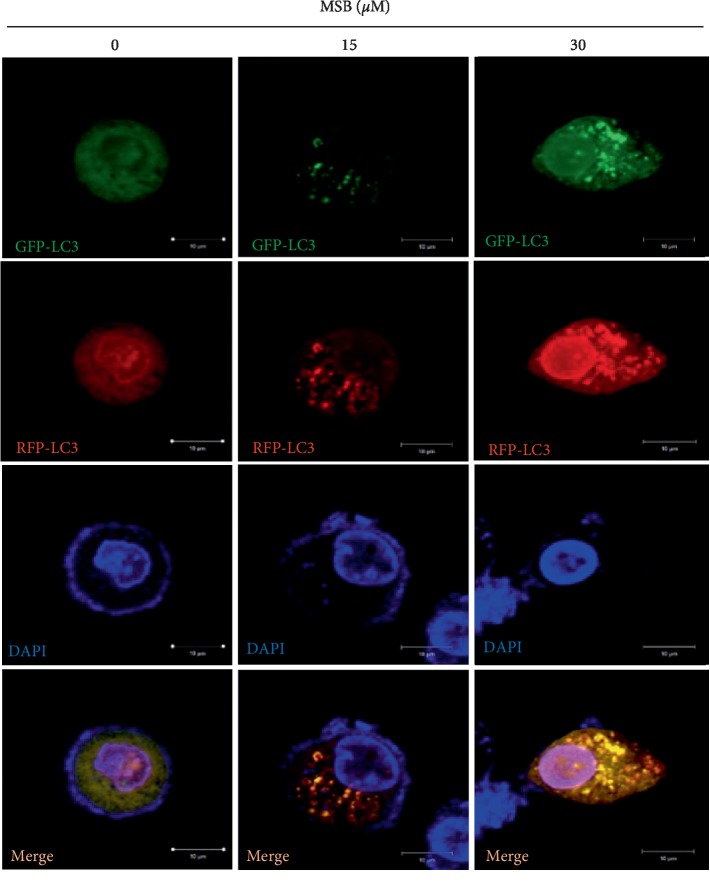
Misaponin B disturbed autophagic flux in A549 cells. After transfection with RFP-GFP-LC3 plasmid, A549 cells were treated by Misaponin B and visualized by confocal microscopy. Representative fluorescence images showing the red/green signals from the mRFP-GFP-LC3 construct for measuring the autophagic flux. Nuclei were stained with DAPI (blue). Punctae: RFP+GFP+ (R+G+: early autophagosomes), RFP+GFP− (R+G−: autolysosomes), and total LC3 punctae. Images merged red and green channels; scale bar: 10 *μ*M (*n* = 2).

To investigate whether or not Misaponin B induces autophagic flux, A549 cells were transfected with a tandem RFP-GFP-LC3 construct and then exposed to Misaponin B (15 and 30 *μ*M) for 24 h. Then, autophagy flux was evaluated in A549 cells by using confocal microscopy analysis. Misaponin B exhibited yellow color punctae by merging image in RFP-GFP-LC3 construct-transfected A549 cells (Figure 4).

## 4. Discussion

The current study was undertaken to elucidate the underlying antitumor mechanism of Misaponin B in A549 and AsPC-1 cancer cells. Herein, Misaponin B exerted cytotoxicity in A549, H460 nonsmall cell lung cancer, SKOV3 ovarian cancer, and AsPC-1 pancreatic cancer cells, implying significant cytotoxic effect of Misaponin B in several cancers.

To check the type of cell death by Misaponin B in A549 and AsPC-1 cells, cell cycle analysis and western blotting were performed. Here, Misaponin B did not induce PARP cleavages up to 40 *μ*M (Supplementary Figure 1), but cell cycle analysis revealed that Misaponin B induced G2/M arrest and no sub-G1 accumulation in A549 and AsPC-1 cells, indicating the cytotoxicity of Misaponin B can be induced by G2/M arrest, not apoptosis signaling, in A549 and AsPC-1 cells.

Autophagy is the catabolic process for the recycling of macromolecules and the degradation of long-lived proteins, pathogens, damaged DNA elements, and damaged organelles [26, 27]. Growing evidences have demonstrated that impaired autophagy by blocking of fusion of autophagosome and lysosome would rather cause cell death due to over consumption of energy to survive [28]. It is well known that autophagic cell death can be induced, only when apoptosis is blocked by caspase inhibitor or lacking Bax and Bak expression in cells [29, 30]. In the current study, Misaponin B increased the conversion of LC3B and green GFP-LC3 punctae as a typical autophagy marker by western blotting, qRT-PCR, and immunofluorescence in A549 cells, demonstrating the potent autophagy induction by Misaponin B in A549 and AsPC-1 cells. Autophagy flux assay is frequently employed to assess autophagy degradation process in the cells [31]. Usually, GFP and mRFP signals are detected by merging image fluorescence before fusion with lysosomes, while only the mRFP signal is detected by merging image red fluorescence after fusion with lysosomes for complete autophagy flux [32–34]. The green-red/yellow punctae indicate autophagosomes, whereas the autolysosome (autophagosome fused to lysosome) would be red in color [35]. Here, autophagy flux assay using GFP-mRFP-LC3 construct transfection showed that Misaponin B increased merged yellow GFP-LC3 punctae in A549 cells, implying that Misaponin B impaired autophagy to induce autophagic cell death in A549 cells. Likewise, previous evidences revealed that arsenic trioxide treatment of malignant glioma cells induced G2/M arrest and autophagic cell death [19], and plumbagin inhibited proliferation to undergo G2/M arrest and autophagic cell death [36]. However, for detailed mechanistic study, further molecular studies including PPI, luciferase assay, and animal study are required with Misaponin B in the near future.

## 5. Conclusions

In summary, Misaponin B exerted significant cytotoxicity, induced G2/M arrest, and increased the expression of LC3B, a maker of autophagy, at the protein and mRNA levels in A549 cells. Furthermore, Misaponin B increased autophagic vacuoles GFP-LC3 punctae in A549 cells, but Misaponin B disturbed autophagy flux by revealing merged yellow GFP-LC3 punctae. Overall, our findings support evidences that Mi-saponin B induces G2/M arrest and autophagic cell death as a potent anticancer agent.

## Data Availability

The data and materials supporting the conclusions of this article are included within the article.
